# Quality of Rye Plants (*Secale cereale*) as Affected by Agronomic Biofortification with Iodine

**DOI:** 10.3390/plants12010100

**Published:** 2022-12-25

**Authors:** Anna Krzepiłko, Bogdan Kościk, Monika Skowrońska, Sebastian Kuśmierz, Jacek Walczak, Roman Prażak

**Affiliations:** 1 Faculty of Food Sciences and Biotechnology, University of Life Sciences in Lublin, Skromna 8, 20-704 Lublin, Poland; 2 Faculty of Economic and Technical Sciences, Pope John Paul II State School of Higher Education in Biała Podlaska, Sidorska 95/97, 21-500 Biała Podlaska, Poland; 3 Department of Agricultural and Environmental Chemistry, University of Life Sciences in Lublin, Akademicka 15, 20-950 Lublin, Poland; 4 National Research Institute of Animal Production, Department of Production Systems and Environment, Krakowska 1, 32-083 Balice, Poland; 5 Institute of Plant Genetics, Breeding and Biotechnology, University of Life Sciences in Lublin, Akademicka 15, 20-950 Lublin, Poland

**Keywords:** potassium iodide, potassium iodate, soil application, foliar application, total antioxidant capacity, photosynthetic pigment

## Abstract

This study assessed the possibility of using iodine-containing fertilizers for agronomic biofortification of rye biomass used as fodder for cows, and establish the best application method and form and the optimal dose of iodine (I) under field conditions. The impact of iodine fertilization on grain iodine content was not studied. Results showed that agronomic biofortification of rye plants with iodine, influenced by its dose, form, and method of application was highly effective in increasing I shoot contents. Plant I-enrichment via foliar and soil application significantly affected I concentration in plant biomass even at a low dose (2.5 kg ha^−1^). Soil I application as KI appeared optimal for rye plants used as fodder for cows, especially cropped under the soil with a neutral reaction. Iodine application improved the biological quality of rye plants by increasing concentrations of sugar, chlorophylls, and at a low rate, protein and total antioxidant capacity.

## 1. Introduction

Iodine (I) is an essential micronutrient for the development and growth of both humans and animals (mainly mammals). This element is the main component of the thyroid hormones (TH), thyroxine (T_4_), and triiodothyronine (T_3_), which contribute to the growth and development of the body, biosynthesis of proteins, and nervous system functions [1,2,3,4]. Its inadequate intake leads to iodine deficiency disorders (IDDs) manifested, among others, in hypothyroidism, a high incidence of goiter, thyroid and stomach cancer, reduced IQ, mental impairment and cretinism in a population, decreased fertility rate, increased risk of miscarriage, fetal abnormalities, and higher neonatal and infant mortality [5,6,7,8]. Despite a significant increase in the number of countries with an adequate iodine intake via table salt fortification, globally, almost two billion people even in the USA, Europe, Australia, or New Zealand still consume an insufficient amount of I [3,4,5,9]. It is mainly related to the geochemical cycle of iodine, which concentrates this element in the oceans and coastal zones and reduces its levels in groundwater and mainland soils. Furthermore, the interaction of iodine with soil organic and inorganic components elevates its fixation, diminishes volatilization, and reduces availability to plants [5,8]. Hence, whereas fish, shellfish, or edible seaweeds are generally rich in iodine, crops, especially grown on inland soils, contain very low levels of this nutrient [5,6,9,10].

The usually recommended strategy for controlling iodine deficiency, i.e., universal salt iodization is sometimes becoming untenable, since it conflicts with other important public health objectives, such as the prevention of hypertension and cardiovascular diseases and voluntary, not mandatory, measures that reduce sodium in food products are applied in some countries [8,9,10,11]. Moreover, the use of iodized salt in food processing is still insufficient in many regions of the world [6,9]. On the other hand, in numerous industrialized countries, milk and dairy products are a vital dietary source of I, contributing 25–70% of its total daily intake, which depends on the I content of cow feed. In this regard, the use of iodine-biofortified cow feed to increase its content as a result of carry-over into the milk seems like a good strategy to improve consumers‘ I supply [3,5]. 

Numerous studies indicated that the native iodine content of plant feedstuffs is low and increased with elevating exogenous iodine content. The effects of the iodine biofortification on the quality and quantity of crop production were variable and dependent on the concentration, chemical form, and production system applied in the conducted experiments [12,13]. Some researchers showed beneficial effects of iodine, including better growth, higher yields, positive changes in the plant tolerance to stress, antioxidant capacity, nitrogen use efficiency, contents of glucose, fructose, and total sugars, while others reported that the applications of iodine caused no response or even had negative effects such as biomass depletion or higher chlorosis and necrosis occurrence [8,10,12,13,14,15,16]. It should be noted that there is very little or no information in the scientific literature regarding iodine biofortification of the principal component of an animal diet in the world, i.e., cereal crops [8,9,13], e.g., wheat, rice, and maize [9], especially cropped for biomass. Iodine accumulates to a greater extent in vegetative organs than in grains since it is transported mainly through the xylem in plants [8].

Therefore, enrichment of cereals with iodine is currently a vital research topic and a practical challenge [9]. Due to one of the highest levels of bioactive substances among cereal species, rye seems to be a suitable candidate for iodine biofortification programs. Rye contains peak concentrations of phytonutrients, vitamins, and amino acids and biofortification with iodine and use for green forage or silage could increase the concentration of this element in the intermediate links of the food chain and help in meeting human iodine requirements. The objectives of this study were to assess the possibility of using iodine-containing fertilizers for agronomic biofortification of rye biomass used as fodder for cows, and establish the best application method and form as well as the optimal dose of iodine under field conditions.

## 2. Results and Discussion

### 2.1. Iodine Content

Iodine is not an essential nutrient for land plants, although in aquatic plants participates in antioxidant metabolism [13]. In the present study, I contents in rye plants were influenced by iodine dose, form, and method of application (Table 1). The native iodine concentration of the rye plants investigated in this study ranged from 0.49 to 0.51 mg kg^−1^ fresh matter (FM), which was in accordance with values reported previously in the literature [9,16]. 

Generally, there was a significant progressive raise in iodine concentrations in plants with increasing application rates, particularly for the soil KI treatments (Table 1). The iodine contents of the plant biomass were affected by the soil and foliar-applied KI and KIO_3_ even at their lowest dose (2.5 kg ha^−1^). Other studies have also shown the dose-dependent increase in I concentration of wheat, rice and maize after foliar and soil application of KIO_3_ and KI at increasing rates [9]. According to Weng et al. [17], iodine concentration in plants increases linearly up to soil I content of approx. 55 mg kg^−1^, after which the rate of uptake declined mainly due to the exceedances the absorption capacity of the soil and I leaching.

The KI treatments caused a significantly higher increase in shoot iodine than the KIO_3_ (Table 1). At the highest iodine dose of 5 kg ha^−1^, its application as KI rise shoot I concentration by more than three- and two-fold for soil and foliar fertilization, respectively, compared to the application as KIO_3_ [4,8,9,18]. The larger uptake of I^−^ compared to IO_3_^−^ was also confirmed by Cakmak et al. [9] and Lawson [4], who stated the smaller risk for toxicity to plants for the IO^3−^ species. The iodide form (I^−^) is known to be more soluble in soils and absorbable at a higher rate by the roots than iodate IO_3_^−^, which has a larger molecular weight and higher valence [4,9,15]. Moreover, a slow absorption rate of IO_3_^−^ is limited by its reduction to I^−^ prior plant uptake [4,13]. Many authors indicate, however, that in field conditions IO_3_^−^ shows better phytoavailability than I^−^ due to the longer residence in the soil environment. The iodide form of iodine is easily fixed in soil organic matter and thus becomes unavailable to plants [4]. Acidic soils rich in aluminum and iron ions additionally reduce its bioavailability [3,4,12,15]. Furthermore, I^−^ is faster leached below the rooting zone and/or evaporated as organic iodides than IO_3_^−^ [4]. In the present study, soil pH = 6.51 favored I^−^ phytoavailability. According to Rakoczy-Lelek et al. [12] mainly soils with neutral reaction provide the best conditions for I mobility. In the conducted experiment apparently, dividing the iodine dose of KI and KIO_3_ contributed to the leaching reduction and additionally increased uptake of I by rye plants. Kashparov et al. [19] conducting research on cereals stated that both plant species and soil type influenced I phytoavailability. Approximately 75% of the variation in grass iodine concentrations was explained by the models with pH and organic matter content as dependent parameters [3].

Both foliar and soil biofortification significantly caused iodine to accumulate in plants. The soil application was, however, more than two-fold higher (Table 1). According to Nascimento et al. [15], Smoleń and Sady [20] absorption of iodine is more efficient via soil enrichment than foliar sprays. Some researchers [5] using isotope _125_I demonstrated that over 80% of the iodine uptaken by plants is transferred to their aerial parts. 

Taking into consideration that a) one glass of milk contributes 57% of the WHO recommended iodine intake for adolescents and adults (150 μg/d) [3], b) the increase in the iodine content of milk to 150–200 µg/L requires lactated animals to be given 60 mg of iodine per day: 4.6 kg of rye fodder per day obtained under rye biofortification with 2.5 kg I/ha provides this I amount. It should be underlined that the iodine content of forages is unlikely to cause iodine overfeeding in contrast to mineral mixes or salt licks [21]. Moreover, iodine compounds in salt licks used outside are readily volatilized and/or leached [22] and organic sources of this element such as biofortified feed are more stable [8]. Agronomic biofortification offers a complementary and affordable method to improve consumers‘ iodine supply. Its ongoing costs are minimal, although support may be necessary to optimize fertilizer application [23].

### 2.2. Photosynthetic Pigment Content and Total Antioxidant Capacity (TAC)

The concentration of chlorophylls is treated as an indicator of the photosynthetic capacity and the health status of plants enhancing crop yield [24,25,26]. In the present study, no visible symptoms of chlorosis or necrotic spots were found in the rye plants which indicated non-toxicity of iodine dose, form, or application method to plants. The content of chlorophyll a and b increased significantly in the rye plants fertilized with both iodine forms compared with the control (Table 2 and Table 3). The method of application had no significant impact on the analyzed parameter. The highest increase in both forms of chlorophyll, a and b, was observed after soil application of 5 kg I ha^−1^ in the form of KI. Some authors [13] noticed a rise in the concentration of chlorophyll after treatment with low doses of I^−^, whereas at high I dosage, Ch_a_ declined and a biomass decrease occurred [13]. Results of the studies presented by Lowson [4] indicated that I application of up to 7.5 kg I ha^−1^ was well tolerated by plants. Phytotoxic symptoms were detected at very early stages of plant development when 15 kg I ha^−1^ was applied. Toxic effects of excessive iodine doses were mainly due to intracellular oxidation of I^−^ to elemental iodine followed by iodination of cellular components such as chlorophylls and carotenoids and decreased carbon dioxide assimilation [4,27]. In the present study, it seems that applying low dividing doses of iodine was a good strategy to avoid I toxicity. As Duborská et al. [28] stated, low concentrations of I^−^ and IO_3_^−^ stimulated barley growth and the impact was more intense when the former form of iodine was applied. Moreover, it should be kept in mind that the negative impacts of high I rates were more evident on grain yield than the vegetative plant parts [9]. The content of chlorophyll a in the rye plants was more than two-fold higher than chlorophyll b. According to Michalczyk and Macura [29], plant tissues usually contain up to three times more chlorophyll a than chlorophyll b.

Carotenoids are tetraterpene pigments present in photosynthetic-, similar to chlorophylls, and also in non-photosynthetic organs such as seeds, roots, and flowers. They are crucial in various physiological processes and served as antioxidants, precursors of vitamin A, or photo-protectors in animal and human organisms [30]. In the present study, total carotenoid content ranging from 2.73 to 3.43 mg g^−1^ FW was not significantly influenced by the tested factors (Table 4), which was in accordance with the results of other researchers [31,32].

The total antioxidant activity measured by ABTS and DPPH assays is presented in Table 5 and Table 6. ABTS method is characterized by a higher reactivity of radicals and a reaction with a broader range of antioxidants. Thus, it provided higher values related to Trolox [1,33], varied between 40.03–68.75 µmol Trolox g^−1^ FW in the present study (Table 5 and Table 6).

In the current research, the biofortification of rye plants with iodine caused significant changes in TAC dependent on its dose, form, and method of application (Table 5 and Table 6). Iodine application in rye agroecosystems affected the TAC of hydrophilic and lipophilic antioxidants measured by the ABTS method and hydrophobic determined according to the DPPH test (Table 5 and Table 6). TAC was significantly higher in rye plants supplied with iodine via soil compared to foliar I application. 

When total antioxidant content was measured by the ABTS and DPPH method, an increase in these parameters fertilized with 2.5 kg·ha^−1^ KI was noted (Table 5 and Table 6). Iodine biofortification increases the concentrations of antioxidants (e.g., phenolic compounds, glutathione, and ascorbic acid) as well as the activity of some enzymatic antioxidants (e.g., superoxide dismutase or catalase) that mitigate the negative impact of reactive oxygen species [1,33,34]. Iodine elevates the antioxidant response in plants at low doses and allows greater resistance against abiotic stress such as heavy metals and salinity [6,13,26]. 

In the conducted studies, no statistically significant differences in the impact of KI and KIO_3_ were found (Table 5 and 6). Other authors, especially in the conditions of field research, also obtained ambiguous results [13].

### 2.3. Sugar and Protein Content

In the present study, I supply positively affected sugar concentration in rye shoots up to 5 kg I ha^−1^ (Table 7). It was in accordance with the results of Blasco et al. [35]. In the current research iodine in the form of KIO_3_, as well as soil I application, had a significant positive effect on sugar content in the rye plants (Table 7). According to Grzelak [36], carbohydrate concentrations depend on the plant species, stage of growth, crop harvest time, and sun exposure, which is linked to photosynthesis and water content. 

Total protein concentration (TP) significantly decreased above the rates of 2.5 kg I ha^−1^ (Table 8). The method of plant I-enrichment had no significant impact on the protein content in rye plants. The application of KI caused a statistically significant increase in protein levels compared to the KIO_3_ form. Rangel et al. [11] also noticed TP rise after KI treatments at low and high levels supplied once or at a split-dose. Apparently, KI was more readily absorbed, and potentially led to the production of secondary metabolites and the concomitant production of the proteins crucial for that metabolism [11].

## 3. Materials and Methods

### 3.1. Site Description and Experimental Design

A field experiment was conducted in Zamość Voivodship (50°42′ N, 23°12′ E) on eutrophic brown soils (silt loam) with the following parameters: pH = 6.51, 19.2 g SOC kg^−1^, 274.0 mg P kg^−1^, 127.1 mg K kg^−1^, and 106.0 mg Mg kg^−1^. The experiment was carried out in a randomized complete block design with three replicates. The experiment scheme included 3 factors: (i) iodine dose (0, 2.5, and 5 kg ha^−1^); (ii) iodine form (KI and KIO_3_); (iii) method of iodine application (soil and foliar). Each treatment plot had 18 m^2^. The spacing between plots and blocks was 1.5 m and 3 m, respectively. The test plant was rye (*Secale cereale*), a variety of Pastar sown at a rate of 5 million seeds per hectare. Rye row spacing was 12.0 cm. Before seed sowing (September), nitrogen, phosphorus, and potassium were applied at levels recommended for the forage variety, taking into account their content in the soil. The second portion of nitrogen and half of the recommended amount of iodine (1.25 and 2.50 kg iodine per hectare) were applied in spring (April). The second half of the iodine was applied in May. The trial plots were drenched with diluted I solutions. The iodine as KIO_3_ and KI was applied to the soil and in the form of a plant spray (0.05%). Plant samples were collected in the earing phase of rye.

### 3.2. Laboratory Tests

The iodine content of the test material was determined following incubation with TMAH (tetramethylammonium hydroxide) by ICP-MS (inductively coupled plasma mass spectrometry) according to PN-EN 15111:2008P [37]. 

The sample was prepared as follows. A 500 mL volume of tellurium standard solution was pipetted into a 10 mL volumetric flask, 5 mL of the extract sample was added, and the flask was filled with water to the 10 mL mark. Following calibration of the apparatus, a solution of a blank sample and then solutions of the test samples were analyzed. The iodine content (*w*) was calculated as iodide in mg/kg of the sample using the following equation:

(1) $$w=\frac{p\cdot F\cdot V}{m}$$

where:

*p*—iodine content in the sample solution, in micrograms per liter.
*F*—dilution factor of the sample solution.
*V*—the volume of extract solution (mL).
*m*—initial sample weight (mg).

Extracts for laboratory analysis were prepared from plant material. The aerial parts of the rye plants were crushed, 100 g samples were washed in sterile water, and 100 cc of sterile water distilled at 4 °C was added, after which the samples were placed in an ice bath and then homogenized for 2 min. The solution was centrifuged for 5 min at 3500 rpm and the supernatant was frozen. Extracts were used to determine protein, reducing sugars, and antioxidant properties.

Protein content was determined by the Lowry method [38] with the Folin-Ciocalteu reagent. The absorbance value was read after 30 min at 750 nm. The protein content of the extracts was determined from a calibration curve.

Miller’s method for determining reducing sugar content using 3,5-dinitrosalicylic acid (DNS) in an alkaline environment at high temperature exploits the reducing properties of sugars [39]. DNS was added to the extracts, the mixture was incubated for 5 min at 90 °C, and sodium potassium tartrate was added. Absorbance was measured at 575 nm. The content of reducing sugars in the extracts was determined on the basis of the calibration curve for glucose.

Determination of total antioxidant capacity by the ABTS (2,2’-azobis (3-ethylbenzothiazoline-6-sulfonic acid)) method involves spectrophotometric measurement of changes in the absorbance of an ABTS^·+^ radical cation solution. Antioxidants present in the extract reduce ABTS^·+^ to ABTS and cause the solution to lose its blue-green colour. A concentrated solution of ABTS^·+^ radical cation was obtained from 2,2’-azino-bis (3-ethylbenzo-thiazoline-6-sulfonic acid) ammonium salt by oxidation with potassium persulfate. The diluted ABTS^·+^ solution was added to the extract. The absorbance of the reaction mixture was measured after 30 min at 414 nm.

Antioxidant activity was calculated as the percent inhibition of ABTS^·+^ radical cation according to the following equation:

(2) $$\%\;\mathrm{inhibition}=100\cdot \frac{A_{0}-A_{c}}{A_{0}}$$

where: *A*_0_—initial absorbance of ABTS^·+^ radical cation; *A_c_*—mean absorbance for the added sample at concentration *c*. Total antioxidant capacity was expressed in Trolox equivalent per g of fresh weight of the aerial parts of the rye plants.

Total antioxidant capacity was determined by the DPPH method. When synthetic DPPH (2,2-diphenyl-1-picrylhydrazyl) radical reacts with antioxidants, it takes on electrons from these compounds and undergoes a color change [40].

A methanol solution of DPPH was added to the extract from the green parts of the rye. The reaction mixture was incubated for 30 min in the dark. The decrease in absorbance was measured at 517 nm [41].

The capacity of the antioxidants in the rye plant extract to counteract the oxidation reaction was calculated according to the following equation [40]:

(3) $$\%\;\mathrm{inhibition}=100\cdot \frac{A_{0}-A_{c}}{A_{0}}$$

where: *A*_0_—mean initial absorbance of DPPH radical solution; *A_c_*—mean absorbance for the added sample at concentration *c*. Total antioxidant capacity was expressed in the equivalent of Trolox (a synthetic antioxidant) per g of fresh weight of the aerial parts of the rye plants.

The total content of carotenoids and chlorophyll a and b was determined by spectrophotometry following extraction with 80% acetone [42]. Absorbance was measured at 663 nm, 646 nm and 470 nm. 

The photosynthetic pigment content of the test sample was calculated according to the equations below and expressed as mg·g^−1^ f.w.

(4) $$\mathrm{chlorophyll}\;a=(12.21A_{663}-2.81A_{646})\cdot \frac{V}{1000\cdot m}$$

(5) $$\mathrm{chlorophyll}\;b=(20.13A_{646}-5.03A_{663})\cdot \frac{V}{1000\cdot m}$$

(6) $$\mathrm{total}\;\mathrm{amount}\;\mathrm{of}\;\mathrm{carotenoids}=({\frac{1000A_{470}-3.27C_{a}-104C_{b}}{229}}_{})\cdot \frac{V}{1000\cdot m}$$

where: *A*—absorbance measured at 663 nm, 646 nm, and 470 nm; *V*—the total volume of extract (cm^3^); *m*—sample weight (g).

### 3.3. Statistical Analysis

A three-way analysis of variance (ANOVA) and Tukey’s mean separation were used to determine the statistical significance at *p* < 0.05. Statistical analyses were performed using Statistica 13.3.

## 4. Conclusions

Agronomic biofortification of rye plants with iodine, influenced by its dose, form, and method of application, was highly effective in increasing I shoot contents. 

Plant I-enrichment via foliar and soil application significantly affected I concentration even at a low dose (2.5 kg ha^−1^). Soil I application as KI appeared optimal for rye plants used as fodder for cows, especially cropped under the soil with a neutral reaction. Iodine application improved the biological quality of rye plants. Concentrations of sugar, chlorophylls, and at a low rate (2.5 kg I ha^−1^), protein and total antioxidant capacity significantly increased as a result of I use. 

## Figures and Tables

**Table 1:** Content of iodine in rye plants depending on I dose, form, and application method.

Iodine Form (A)	Application Method (B)	Iodine Dose (C)			Mean (A)
		0	2.5	5	
		(mg kg^−1^)			
KI	Soil	0.50f	13.11b	20.24a	7.99A
	Foliar	0.51f	7.90c	5.70d	
KIO_3_	Soil	0.49f	4.94d	5.33d	
	Foliar	0.50f	2.04e	2.42e	1.65B
	**Mean (C)**	0.50C	7.00B	8.42A	
	**Mean (B)**	Soil: 7.44A	Foliar: 3.18B		

The same letter means not significantly different. Uppercase letters compare means of iodine form, application method, and iodine dose. Lowercase letters compare means between treatments.

**Table 2:** Content of chlorophyll in rye plants depending on I dose, form, and method of application.

Iodine Form (A)	Application Method (B)	Iodine Dose (C)			Mean (A)
		0	2.5	5	
		(mg g^−1^ Fresh Weight)			
KI	Soil	9.81e	10.00de	13.95a	11.88A
	Foliar	10.12de	13.97a	13.44a	
KIO_3_	Soil	10.01de	12.82a	12.04abc	
	Foliar	9.98de	10.91cde	11.15bcd	11.15B
	**Mean (C)**	11.44C	11.92B	12.65A	
	**Mean (B)**	Soil: 11.44_A_	Foliar: 11.59_A_		

The same letter means not significantly different. Uppercase letters compare means of iodine form, application method, and iodine dose. Lowercase letters compare means between treatments.

**Table 3:** Content of chlorophyll b in rye plants depending on I dose, form, and method of application.

Iodine Form (A)	Application Method (B)	Iodine Dose (C)			Mean (A)
		0	2.5	5	
		(mg g^−1^ Fresh Weight)			
KI	Soil	3.94e	4.63bcde	6.23a	5.18A
	Foliar	4.36de	5.89ab	6.00a	
KIO_3_	Soil	3.91e	5.51abc	5.44abc	4.80B
	Foliar	4.06de	4.95bcd	4.95bcd	
	**Mean (C)**	4.07B	5.25A	5.66A	
	**Mean (B)**	Soil: 4.94_A_	Foliar: 5.03_A_		

The same letter means not significantly different. Uppercase letters compare means of iodine form, application method, and iodine dose. Lowercase letters compare means between treatments.

**Table 4:** **C**arotenoids content in rye plants depending on I dose, form, and method of application.

Iodine Form (A)	Application Method (B)	Iodine Dose (C)			Mean (A)
		0	2.5	5	
		(mg g^−1^ Fresh Weight)			
KI	Soil	2.73a	3.03a	3.15a	3.20A
	Foliar	3.64a	3.23a	3.43a	
KIO_3_	Soil	3.32a	3.25a	3.17a	3.19A
	Foliar	3.22a	2.98a	3.21a	
	**Mean (C)**	3.23A	3.12A	3.24A	
	**Mean (B)**	Soil: 3.11_A_	Foliar: 3.29_A_		

The same letter means not significantly different. Uppercase letters compare means of iodine form, application method, and iodine dose. Lowercase letters compare means between treatments.

**Table 5:** Total antioxidant capacity (TAC ABTS) in rye plants depending on I dose, form, and method of application.

Iodine Form (A)	Application Method (B)	Iodine Dose (C)			Mean (A)
		0	2.5	5	
		(µmol Trolox g^−1^ Fresh Weight)			
KI	Soil	40.03ef	68.75a	55.08c	48.72_A_
	Foliar	41.31e	48.52d	38.64f	
KIO_3_	Soil	40.44ef	61.07b	40.55ef	
	Foliar	40.60ef	40.29ef	53.61c	46.09_B_
	**Mean (C)**	40.59C	54.66A	46.97B	
	**Mean (B)**	Soil: 50.98A	Foliar: 46.09B		

The same letter means not significantly different. Uppercase letters compare means of iodine form, application method, and iodine dose. Lowercase letters compare means between treatments.

**Table 6:** Total antioxidant capacity (TAC DPPH) in rye plants depending on I dose, form, and method of application.

Iodine Form (A)	Application Method (B)	Iodine Dose (C)			Mean (A)
		0	2.5	5	
		(µmol Trolox g^−1^ Fresh Weight)			
KI	Soil	4.20e	5.19a	4.87ab	4.50A
	Foliar	4.22de	4.28de	4.23de	
KIO_3_	Soil	4.32de	4.67bc	4.65bcd	
	Foliar	4.15e	4.42cde	4.31de	4.42A
	**Mean (C)**	4.22B	4.64A	4.51A	
	**Mean (B)**	Soil: 4.65A	Foliar: 4.27B		

The same letter means not significantly different. Uppercase letters compare means of iodine form, application method, and iodine dose. Lowercase letters compare means between treatments.

**Table 7:** Content of sugar in rye plants depending on I dose, form, and method of application.

Iodine Form (A)	Application Method (B)	Iodine Dose (C)			Mean (A)
		0	2.5	5	
		(mg g^−1^ Fresh Weight)			
KI	Soil	0.29a	0.39a	0.42a	0.35B
	Foliar	0.29a	0.33a	0.36a	
KIO_3_	Soil	0.31a	0.43a	0.42a	0.37A
	Foliar	0.3a	0.39a	0.39a	
	**Mean (C)**	0.30B	0.39A	0.40A	
	**Mean (B)**	Soil: 0.38A	Foliar: 0.34B		

The same letter means not significantly different. Uppercase letters compare means of iodine form, application method, and iodine dose. Lowercase letters compare means between treatments.

**Table 8:** Content of protein in rye plants depending on I dose, form, and method of application.

Iodine Form (A)	Application Method (B)	Iodine Dose (C)			Mean (A)
		0	2.5	5	
		(mg g^−1^ Fresh Weight)			
KI	Soil	1.46f	2.18a	1.79cd	1.84A
	Foliar	1.54ef	2.12ab	1.92ac	
KIO_3_	Soil	1.49f	1.68def	1.73cde	1.62B
	Foliar	1.51ef	1.74cde	1.59def	
	**Mean (C)**	1.50C	1.93A	1.76B	
	**Mean (B)**	Soil: 1.72A	Foliar: 1.74A		

The same letter means not significantly different. Uppercase letters compare means of iodine form, application method, and iodine dose. Lowercase letters compare means between treatments.

## Data Availability

Not applicable.
